# Practical recommendations for the implementation of shared decision-making in multiple myeloma care

**DOI:** 10.1371/journal.pone.0355283

**Published:** 2026-08-28

**Authors:** Jolien Broekmans, Elise Schoefs, Charlotte Verbeke, Lien De Proost, Silène ten Seldam, Eilidh Duncan, Ariel Aviv, Varda Shoham, Michel Delforge, Anneleen Vanhellemont, Isabelle Huys

**Affiliations:** 1 Department of Pharmaceutical and Pharmacological Sciences, KU Leuven, Leuven, Belgium; 2 Myeloma Patients Europe, Brussels, Belgium; 3 Department of Hematology, Ha’Emek Medical Center, Afula, Israel; 4 AMEN Association, Kiron, Israel; 5 Department of Hematology, University Hospitals Leuven, Leuven, Belgium; Tekirdag Namik Kemal University: Tekirdag Namik Kemal Universitesi, TÜRKIYE

## Abstract

**Background:**

Multiple myeloma (MM) is characterized by complex and evolving treatment pathways, involving multiple preference-sensitive decisions across the disease trajectory. Shared decision-making (SDM) is widely recognized as a best-practice approach for such decisions, yet practical, context-specific guidance for implementing SDM in MM care remains limited.

**Objective:**

To develop a set of practical, stakeholder-informed recommendations to support the implementation of SDM in MM care.

**Methods:**

This study was conducted as part of a European mixed-methods research project initiated by the patient organization Myeloma Patients Europe. Building on empirical studies involving MM patients and healthcare professionals (HCPs), an initial set of 24 recommendations was developed. These recommendations were subsequently refined, and agreed upon through two online multi-stakeholder workshops, involving patients, carers, hematologists, oncology nurses, and patient organization representatives from multiple European countries. Qualitative workshop data were thematically analyzed, and recommendations were consolidated through iterative team discussions.

**Results:**

The final output comprises ten core recommendations addressing SDM implementation at multiple levels. Key themes included in the recommendations are adopting a personalized and preference-sensitive approach to patient involvement, tailoring information provision, openly discussing all treatment options including no treatment, strengthening multidisciplinary communication, supporting patient preparation and partnership, ensuring continuity of care, and providing structural, educational, and organizational support for SDM.

**Conclusions:**

These recommendations translate SDM principles into guiding considerations tailored to the specific challenges of MM care. They provide a foundation for context-sensitive implementation efforts aimed at strengthening patient-centered decision-making in MM.

## Introduction

Multiple myeloma (MM) is a hematological malignancy characterized by complex treatment and care pathways, and much variation in disease trajectories across individuals and countries [1,2]. The disease is incurable; it typically follows a relapsing-remitting course [3,4]. Therapeutic advances have expanded the number of available treatment options including stem cell transplantation, immunotherapy, and chemotherapy [5,6]. Although these developments have improved survival outcomes, they have also introduced a growing set of trade-offs for patients related to quality of life and treatment burden [7]. MM patients are confronted with multiple, often preference-sensitive decisions throughout their disease trajectory [4]. As they navigate these decisions, MM care requires not only clinical expertise but also high-quality communication processes that accommodate patients’ evolving values and preferences [6].

For such preference-sensitive decision trajectories, shared decision-making (SDM) is widely recognized as a best-practice approach [2,6,8]. SDM is a collaborative process in which patients and healthcare professionals (HCPs) make decisions together based on the best available evidence and patients’ values and preferences [8,9]. However, the MM context introduces some specific layers of complexity for the implementation of SDM. Newly diagnosed patients often experience emotional overload and cognitive fatigue, which may limit their ability to process information and articulate preferences, particularly if not elicited [10]. Moreover, patients’ preferences may shift substantially over the course of the disease as their clinical condition, treatment experiences, knowledge, and personal circumstances evolve [11–13]. From the HCP perspective, structural barriers such as time pressure, both system-related and in emergency situations, rapidly evolving treatment landscapes, and the multitude of decision points can constrain opportunities for high-quality communication and preference exploration [14].

Although many of the above-mentioned challenges mirror those described in the broader oncology literature [15], MM care does represent both a high-priority and a high-complexity setting for implementing SDM. Yet, practical, context-specific and stakeholder-informed guidance for embedding SDM within MM care remains limited. To address this gap, the present paper reports on the development and evaluation of a set of practical recommendations for implementing SDM in MM care. These recommendations build on findings from preceding empirical research involving MM patients and HCPs [16,17], and were subsequently refined, consolidated, and agreed upon through multi-stakeholder workshops including patients, carers, hematologists, oncology nurses, and representatives of patient organizations. Although the focus in this paper is on MM, the developed recommendations may also be applicable to support SDM in other disease contexts involving preference-sensitive and value-driven treatment decisions.

In what follows, we first outline the mixed-methods approach underpinning the development of the recommendations. We then present the final set of recommendations. Lastly, we reflect on implementation challenges and offer some additional remarks.

## Methods

The recommendations presented in this paper are developed as part of a research project that is initiated and supported by the patient organization Myeloma Patients Europe [18], and focuses on the implementation of SDM in MM care. Throughout the whole research process, a project steering committee was closely involved. The committee comprised MM patients (n = 3), hematologists (n = 3), researchers (n = 2), and a carer (n = 1). Committee members contributed to the formulation of the research questions, advised on study design and methodology, and critically reviewed the draft recommendations and study findings.

**Fig 1:**
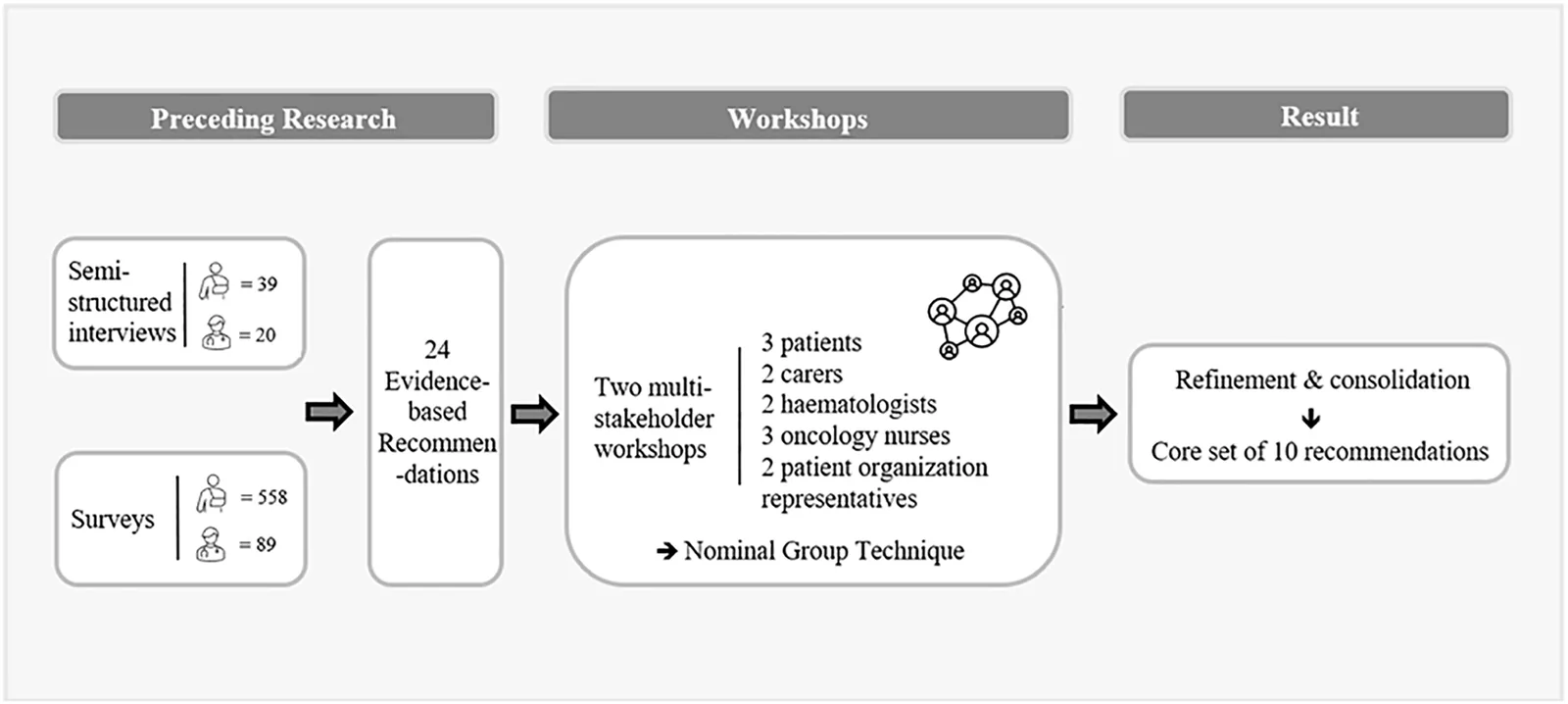
Overview of the mixed-methods study design of the whole research project.

The project adopts a mixed-methods design comprising multiple sequential study phases: a qualitative interview study, a quantitative survey study, and multi-stakeholder workshops (see Fig 1 for an overview of the study design).

As a first step in the project, a qualitative interview study was conducted to explore current SDM practices in MM care and identify key barriers to, and facilitators of, SDM implementation [16]. Subsequently, a quantitative survey study was performed to systematically assess the perspectives of MM patients and HCPs on SDM and quantify elements of SDM and patient involvement [17]. Building on these findings, a preliminary set of 24 recommendations was developed (see S1 Table). To enhance clarity and applicability, the recommendations were organized on three levels: (i) the HCP level, (ii) the patient level, and (iii) the organizational level. The recommendations were then refined, and agreed upon through two multi-stakeholder workshops. The workshops were conducted online in March 2024. Ethical approval for this project was obtained from the Ethics Committee Research UZ/KU Leuven, Belgium (S66580).

English-speaking stakeholders were identified through the research team’s network, including collaborating national patient organizations and participants from the interview study in the first phase of the project. They were contacted via email between 15th of February 2024 and 15th of March 2024. Purposive sampling was used, based on the researchers’ judgement of individuals’ ability to contribute relevant expertise or lived experience relevant to SDM in MM. The sampling strategy aimed to achieve representation from all predefined stakeholder groups and multiple European countries (see Table 1 for the participant information). Each workshop could include participants from the following groups: (i) MM patients, (ii) myeloma patient organization representatives, (iii) carers, (iv) hematologists, and (v) oncology nurses. Furthermore, convenience sampling was applied to account for participants’ availability to attend one of the scheduled workshops. The aim was to include at least one participant for each stakeholder group from various countries across the workshops. Prior to the workshops, all participants received the 24 draft recommendations to allow for preparation and reflection. Right before the start of the workshops, informed verbal consent was obtained from all participants. Consent was documented in the participant list and witnessed by the research team present during the workshops.

**Table 1:** Distribution of stakeholder groups and countries across the workshops.

Workshop 1		Workshop 2	
**Stakeholder group**	**Country**	**Stakeholder group**	**Country**
*Nurse*	Belgium	*Nurse*	United Kingdom
*Nurse*	Germany	*Hematologist*	Belgium
*Patient*	The Netherlands	*Hematologist*	France
*Patient*	Serbia	*Patient organization*	Belgium
*Patient*	Luxembourg	*Patient organization*	Hungary
*Carer*	Germany	*Patient*	Israel

The workshops followed the Nominal Group Technique [19], a structured consensus-building method. Given the multidisciplinary nature of SDM in MM care, this method enabled stakeholders with different backgrounds to discuss and prioritize recommendations while ensuring that all individuals had the opportunity to contribute. During the sessions, each recommendation was presented and explained, after which participants were invited to provide feedback on both content and wording, and rank the recommendations on highest importance. Qualitative data generated during the workshop discussions were analyzed using framework analysis in NVivo, allowing systematic comparison and synthesis of stakeholder perspectives [20].

Following the workshops, the initial categorization across the three levels was omitted, as the research team determined that SDM is best addressed through a holistic, cross-level approach, with several recommendations spanning multiple levels simultaneously. Through iterative discussions among the researchers of the project, the initial 24 recommendations were synthesized into a final set of ten core recommendations by grouping recommendations addressing overlapping themes, generalizing where appropriate, and removing recommendations that were largely redundant or sufficiently represented within broader recommendations.

## Results

Table 2 lists ten core recommendations, consolidated from the full set based on thematic overlap and stakeholder input. Below, we provide background on each recommendation, describe its empirical basis, and include some supporting or illustrative quotes from the workshops. It is important to note that the recommendations are not presented in a specific order.

**Table 2:** Final set of recommendations.

• Adopt a **personalized and preference-sensitive approach to patient involvement** throughout the disease trajectory • Provide **tailored, accessible, and comprehensible information** to support decision-making • Integrate **psychosocial support** as an essential component of SDM • Discuss **all clinically appropriate options** openly and allow **time for deliberation** • Promote **equal and collaborative communication** within and across the multidisciplinary team (MDT) • Encourage **patient preparation and empowerment** to support active participation in consultations • Support a shift towards **patient partnership** and create **awareness about SDM** • Ensure **continuity of care** and **strengthen the role of nurses** • Provide **structural** and **educational support at the organizational level** to enable SDM • Develop **accessible educational resources** and **patient decision aids** tailored to MM

### 1. Adopt a personalized and preference-sensitive approach to patient involvement throughout the disease trajectory

Patients’ desire to be involved in treatment decisions was found to be highly individual and context dependent. Interview and survey findings demonstrated that emotional state, disease stage, and prior treatment experiences shape patients’ preferred level of involvement, which may change over time. This variability was also emphasized during the workshops. As one nurse noted, *“Patients do not want to be involved in the same way. The trajectory of patients over time may change in terms of the level of decision-making”* (Nurse, WS2). From the patient perspective, this sometimes translates into personalized needs that must be met before the patient can be involved. Participants highlighted that for some patients it is particularly important to be given sufficient space to ask questions and engage in dialogue. One patient stressed that *“Patients would like to (…) have more information and to have time to ask a lot of questions, and to have equal communication with the hematologist”* (Patient, WS1). These insights underscore the need for HCPs to actively and repeatedly explore patients’ desired level of involvement and needs and explicitly inquire about treatment preferences and previous experiences, particularly at critical decision points (e.g., stem cell transplantation (SCT) decisions), rather than making assumptions or implicitly steering decisions. Example questions that can be asked to determine a patient’s preferred level of involvement, after the patient has received information, are: *“Now that we have discussed all the different options, are there aspects of your life that you think are important to consider when making this decision? Are there characteristics of treatments that you do (not) like?”.*

### 2. Provide tailored, accessible, and comprehensible information to support decision-making

Clear and understandable information was consistently identified as a core component of SDM. However, many patients from the interviews and survey reported that their information preferences were insufficiently explored and that understanding was not always checked. Workshop participants emphasized that preferences regarding the type, amount, format, and timing of information vary widely and can differ across ages or familiarity with information sources. As one nurse explained, *“Some patients want to have very in-depth information. Others don’t want to have at all, very in-depth information”* (Nurse, WS2). One patient noted that “*Older people perhaps need support in booklets whereas younger people don’t, they’re more used to looking at their phones”* (Patient, WS1). These findings illustrate the importance of adapting information provision to individual patients and explicitly attending to comprehension at each decision point. Example questions that can be asked to determine a patient’s preferred type of information and understandability are: *“Did you fully understand everything? Maybe we can once more repeat the key points?”, “Do you want to receive any additional information regarding your options?”, “What type of information would you like to receive? (reading material, videos, patient leaflets, information provided by patient organizations, or from other sources)”.*

### 3. Integrate psychosocial support as an essential component of SDM

A diagnosis of MM was frequently described as overwhelming, leaving some patients emotionally unable to meaningfully engage in decision-making. Across the interviews and surveys, addressing emotional distress, fears, and concerns was identified as a key facilitator of SDM. Participants emphasized that decision-making cannot be reduced to information exchange alone. As one carer stated, *“It’s really important that the patient feels that [the HCP] is having a holistic approach”* (Carer, WS1). These insights highlight the need for HCPs to explicitly attend to patients’ psychosocial needs alongside clinical discussions and to ensure timely access to appropriate psychological or supportive care when required. One way of providing this psychosocial support is using the holistic needs assessment questionnaire [21] which is designed to identify and assess the needs and concerns of people living with cancer. If necessary, HCPs or the MDT should refer patients to psychologists, social workers, or other professionals with relevant disease knowledge who can offer emotional or social support. Although the availability of psychosocial support varies across European countries, it is important for all to allocate sufficient resources and give greater priority to its provision.

### 4. Discuss all clinically appropriate options openly and allow time for deliberation

Participants indicated that treatment decisions in MM are often made under time pressure, which can limit opportunities for reflection and meaningful patient involvement. In addition, the option of no or discontinued treatment was not always explicitly discussed, despite its relevance in certain situations. During the workshops, patients described positive experiences when alternative options were openly addressed. One patient recounted, *“I said, ‘I hope you don’t mind, but I’d like to not do maintenance treatment,’ and she said, fine”* (Patient, WS1). Such examples illustrate the value of open dialogue about all clinically appropriate options, including treatment deferral, discontinuation, or palliative care where relevant. If palliative care is chosen, patients should be guided in this process by their hematologist and specialized HCP. Allowing patients sufficient time to deliberate, whenever clinically feasible, can support more preference-sensitive and informed decision-making. When there is no urgency, follow-up conversations or support from other HCPs such as nurse specialists should be available to allow patients time to consider and process the information they have received, or to access additional information.

### 5. Promote equal and collaborative communication within and across the multidisciplinary team

Barriers to SDM were identified at both, the patient and MDT level, including power imbalances, inconsistent communication, and limited information sharing within MDTs. Participants emphasized that open, respectful communication is essential for enabling patients to express their preferences. As one patient noted, *“If your physician is very open, and he has got the time, the patient will let you know everything you need, if you ask”* (Patient, WS2). Beyond individual consultation, carers stressed the importance of coordination across professionals. One participant stated, *“The networking and communication between the different doctors I think is so important”* (Carer, WS1). These findings underline the need for MDTs to actively reduce power imbalances and ensure that patients’ preferences, information needs, and psychosocial concerns are consistently shared across team members. Patients’ information or preferences can for example be discussed during the multidisciplinary oncology meetings, if applicable, or noted in a specific section in the medical health record. This ensures that each member can contribute valuable insights from their respective specialties to aid in decision-making.

### 6. Encourage patient preparation and empowerment to support active participation in consultations

Patients from the interviews described difficulties remembering information and formulating questions during consultations. Preparing in advance was viewed as a way to empower patients, facilitate more balanced conversations, and improve the use of consultation time. One patient emphasized the importance of preparation, stating, *“For me, patient preparation is very important (…), I put it even in the first place”* (Patient, WS2). Similarly, a patient highlighted that *“Patients need to be well prepared to have information and then to have time to think about it”* (Patient, WS1). Encouraging patients to reflect on their questions, preferences, and concerns beforehand, potentially supported by prompt question lists or decision aids, can therefore strengthen their participation in decision-making. Examples of questions that patients can prepare for their consultation are: *“What are my options?”, “What are the pros and cons of each option for me?”, “What practicalities are associated with each option?”, “How do I feel about these pros and cons?”, “What is important to me in my treatment?”, “How do my personal preferences relate to my options?”.* Additionally, hospitals can support patient empowerment by placing flyers or showing slides on hospital screens, prompting patients to ask specific questions to initiate the SDM process.

### 7. Support a shift towards patient partnership and create awareness about SDM

Some patients from the interviews and survey expressed reluctance to participate in decision-making due to a lack of confidence, habitual deference to clinicians, or fear of making the wrong decision. At the same time, participants emphasized that meaningful SDM requires a broader cultural shift in how patient roles are perceived. As one nurse noted, *“The mindset needs to change in some countries or in some hospitals”* (Nurse, WS1). These findings suggest that fostering patient partnership involves not only supporting patients in recognizing their right to participate, but also encouraging HCPs to view patients as partners in care, while remaining attentive to individual preferences for more or less involvement. This shift in HCPs’ mindset can be achieved by for example raising awareness regarding SDM through workshops given by patient organizations or key opinion leaders in the field of MM, increase presentations on this topic at congresses in disease-specific sessions to enhance awareness and discussion among HCPs, increase research efforts regarding SDM in MM, and implement professional training in SDM across specialties and curricula. At the level of the patient, a shift in mindset can for example be prompted by patient organizations educating MM patients on the SDM concept, steps, and its evidence-based practices, making patients aware of the possibility of involvement and empowering them to actively participate in decision-making.

### 8. Ensure continuity of care and strengthen the role of nurses

Continuity of care and the role of nurses emerged as key facilitators of SDM. Nurses were described as accessible points of contact who support understanding, provide reassurance, and relay information within the care team. As one nurse explained, *“We are that continuity. We are that point of contact”* (Nurse, WS2). From the patient perspective, frequent changes in care providers were experienced as disruptive to trust and communication. One patient noted, *“If you see another person every month, it’s not the most ideal… that would be ideal, one doctor and one nurse”* (Patient, WS2). These insights highlight the importance of designated points of contact and investment in the nursing role to support SDM across the disease trajectory. Patients should have a single point of contact whose contact details are provided immediately after the first consultation. A more prominent role for the nurse can be achieved by providing additional resources and training to maximize their impact. Potential roles of other members of the MDT (e.g., psychologist, general practitioner, cardiologist, orthopedist) should also be considered.

### 9. Provide structural and educational support at the organizational level to enable SDM

Participants identified multiple organizational barriers to SDM, including time pressure, lack of privacy, insufficient staffing, and limited training opportunities. Structural support from hospital management was considered essential for sustainable implementation. In particular, training opportunities to improve communication skills were repeatedly emphasized. As one carer stated, *“Communication skills, bedside manner… that’s all part of the practice”* (Carer, WS1). In addition, participants stressed that SDM training should be embedded structurally rather than offered on a voluntary basis. One nurse argued that *“Shared decision-making training should not be optional”* (Nurse, WS1). Together, these insights point to the need for organizations to allocate sufficient resources, consultation time, and infrastructure, and to integrate mandatory SDM and communication training into professional education. Specific examples of how the hospital can structurally support SDM is to foresee additional time for consultations, having specific time blocks for consultations where hematologists can fully concentrate on the patient without being disturbed, and providing private consultation rooms. Training for HCPs should be hands-on following a practical guide with case studies including examples of patients not wanting to be involved and feeling overwhelmed, together with social and cultural issues that can come up during consultations. Collaboration with renowned specialists in the MM field that can serve as role models for SDM would be valuable.

### 10. Develop accessible educational resources and patient decision aids tailored to MM

A lack of accessible and objective educational materials for patients was identified as a barrier to SDM. Participants highlighted the value of educational resources and patient decision aids that are evidence-based, co-developed with patients and HCPs, and tailored to MM-specific decision points. Participants also noted that such resources may represent relatively feasible implementation steps. One nurse described them as *“Quick wins”* (Nurse, WS2). Developing patient-friendly materials and decision aids can therefore support informed and preference-sensitive decision-making across the care trajectory. Such patient materials should contain information about for example the disease and possible treatments including their (dis)advantages, risks, outcomes, and practical aspects such as treatment modalities and regimens. Materials should be available in native languages of patients, written in lay language, and tailored towards the treatment options available in the relevant country. As one patient advocate emphasized, *“They should be available in the native language of the patients, written in lay-language”* (Patient advocate, WS1). The materials should be disseminated via various sources (HCPs, patient organizations) using different channels (oral discussion, gatherings, information leaflets, webinars, educational videos, podcasts, and websites).

## Discussion

The recommendations in this paper translate SDM principles into guiding considerations tailored to the complex and evolving context of MM care [2,22,23]. Several considerations, however, warrant discussion regarding their application and implementation in clinical practice.

The set of recommendations may be relevant beyond MM and could inform SDM implementation in other disease contexts. Yet, it is important that it is understood as a best practice guide rather than a rigid protocol. Also, the applicability, feasibility, and relevance of individual recommendations may vary across countries and healthcare systems, reflecting differences in resources, professional roles, and the maturity of SDM implementation [16,24–27]. Rather than undermining their value, this variation highlights the importance of context-sensitive and phased implementation. Furthermore, the recommendations are interdependent and mutually reinforcing. For example, encouraging patient preparation is unlikely to be effective without adequate information provision, time for deliberation, and organizational support [16,28]. Similarly, personalized patient involvement requires not only clinician communication skills but also continuity of care and a supportive multidisciplinary environment [16,29]. This underscores the need for a coherent, system-level approach to SDM implementation.

While some recommendations can be perceived as relatively easy to implement, others raise concerns regarding feasibility, particularly in relation to resource constraints. Indeed, several system-level and organizational conditions are prerequisites for meaningful implementation: SDM for MM necessitates sufficient consultation time, opportunities for follow-up discussions, and space for patients to reflect on information [9,12]. Therefore, it may not always be feasible, for example, to apply SDM in MM care during acute situations where immediate treatment is required. In addition, skills and training are critical. SDM requires specific communication competencies, including the ability to convey complex information in accessible ways, elicit patient values, and address emotional responses [12,30]. These findings underscore that SDM does not occur automatically but requires targeted training and ongoing professional development for HCPs [6,30]. Organizational infrastructure and support from hospital management is also essential for sustainable implementation. Structural factors such as staffing levels, privacy, documentation systems, and care coordination play a decisive role in enabling or constraining SDM [24,31,32].

Responsibility for SDM implementation does not rest with HCPs alone [24,33]. While HCPs play a central role in communication and preference elicitation, nurses are key to continuity of care and ongoing patient support [12,16,34]. Healthcare organizations are responsible for creating the structural conditions that enable SDM, including time, training, and resources [24,31,35]. Patient organizations and researchers can contribute by developing educational materials, decision aids, and awareness-raising initiatives [16,36]. Clarifying and aligning these roles is essential to avoid fragmented or inconsistent implementation.

As a possible next step, these recommendations can be further translated into concrete actionable guidance by applying them to an implementation tool like the Action, Actor, Context, Target, Time (AACTT) framework to be able to specify the aimed behavior changes in the MM healthcare setting [37].

### Strengths and limitations

A major strength of this study is its mixed-methods design and the active involvement of diverse stakeholders throughout the research process, enhancing the relevance and credibility of the recommendations. Nevertheless, several limitations should be considered. Participation in multi-stakeholder workshops was limited to English-speaking stakeholders, which could have influenced the diversity of the group of participants. Additionally, the representation of stakeholder groups in each workshop was not optimal, as not every workshop contained at least one participant of each stakeholder group. Although the NGT facilitated balanced participation and efficient consensus building, the findings reflect the perspectives of the study participants and are not necessarily representing consensus among all MM care stakeholders. Furthermore, prioritization of the recommendations was attempted during the workshops, but the feedback of participants was that this was not feasible, as all recommendations were considered equally important. Therefore, the recommendations were consolidated into ten core recommendations and are presented in no specific order. In addition, while the recommendations are grounded in empirical data and stakeholder input, their real-world impact remains to be evaluated in MM care trajectories. Future research should examine how these recommendations can be operationalized and integrated into routine MM care, including the development and evaluation of training programs and decision aids.

## Conclusion

This paper presents a set of multi-level recommendations to support the implementation of SDM in MM care. Building on empirical evidence, the recommendations translate SDM principles into guiding considerations tailored to the complex and evolving context of MM. Together, these recommendations offer a starting point for future implementation efforts aimed at improving patient-centered care in this complex clinical setting. Yet, it is also clear that this will require coordinated action across multiple levels of the healthcare systems.

## Supporting information

S1 Table Overview of the 24 recommendations. (DOCX)
