# Supplementary material for: Practical recommendations for the implementation of shared decision-making in multiple myeloma care
Source: PLoS One. 2026 Aug 28;21(8):e0355283. doi: 10.1371/journal.pone.0355283 (PMC13524330; doi:10.1371/journal.pone.0355283)
Supplement: Supplementary file 1 [file pone.0355283.s001.docx]

**S1 Appendix I.** Overview of the 24 recommendations

| **Level** | **Recommendation** | **Practical implementation** |
| --- | --- | --- |
| **HCPs** | Personalized approach to patient involvement | Assess and regularly re-assess patients’ desired level of involvement in decision-making |
|  | Explicit preference inquiry | Explicitly inquire about patients’ treatment preferences and prior experiences |
|  | Personalized approach of providing information | Tailor the type, amount, format, and timing of information to patients’ preferences and ensure comprehension |
|  | Psychosocial support | Explicitly address patients’ emotional wellbeing, fears, and concerns alongside information provision |
|  | Open dialogue about no treatment | Discuss the option of no (or discontinued) treatment, including palliative care, when relevant |
|  | Time for deliberation | Provide patients with sufficient time to reflect on information and decisions whenever clinically feasible |
|  | Additional support when necessary | Identify patients requiring extra support due to low health literacy, language barriers, cognitive limitations, or socio-economic factors, and adapt communication accordingly |
|  | Assessment of family member involvement | Explore and respect patients’ preferences regarding the involvement of family members in decision-making |
|  | Enhanced communication between the MDT | Share patients’ preferences, information needs, and psychosocial concerns across the MDT |
|  | Equalized communication | Actively reduce power imbalances to enable patients to express views, questions, and preferences |
|  | Shift in mindset of healthcare providers | Foster a culture in which HCPs view patients as equal partners in decision-making |
| **Patients** | Patient preparation | Encourage patients to prepare questions and reflect on preferences prior to consultations |
|  | Shift in patient mindset | Support patients in recognizing their right and role to participate in decision-making, while respecting those who prefer not to be involved |
| **Organization** | Continuity of care | Ensure patients have a designated point of contact to coordinate care and communication |
|  | Language assistance | Provide professional translation services for patients who do not speak the primary language |
|  | Cultural sensitivity training | Integrate cultural sensitivity training into education for hematologists and nurses |
|  | Patient empowerment | Implement organizational actions that actively encourage and normalize patient participation in decision-making |
|  | Educational resources | Provide accessible and objective, patient-friendly educational materials in lay language about MM treatments and decision points |
|  | Creating awareness | Increase awareness of SDM among patients, HCPs, and other stakeholders |
|  | Patient decision aid development | Develop evidence-based, decision point-specific patient decision aids in collaboration with patients |
|  | Professional training | Integrate mandatory SDM and communication training into professional education |
|  | Structural support hospital | Provide organizational resources such as time, staffing, infrastructure, and privacy to support SDM |
|  | Enhanced role of oncology/hematology nurses | Strengthen and resource the role of nurses in supporting SDM across the care trajectory |
|  | Development of a best practice guide for SDM in MM | Establish a practical best practice guide to support consistent SDM implementation |
